# Two Distinct Genotypes of *Spissistilus festinus* (Say, 1830) Reproduce and Differentially Transmit Grapevine Red Blotch Virus

**DOI:** 10.3390/insects14100831

**Published:** 2023-10-23

**Authors:** Madison T. Flasco, Marc F. Fuchs

**Affiliations:** Plant Pathology and Plant-Microbe Biology, School of Integrative Plant Science, Cornell University, Geneva, NY 14456, USA; mf13@cornell.edu

**Keywords:** acquisition, genotype, *Geminiviridae*, *Grablovirus*, *Spissistilus festinus*, transmission

## Abstract

**Simple Summary:**

Two distinct genetic groupings of the treehopper, *Spissistilus festinus* i.e., California (CA) and Southeastern (SE), have been described but it is unknown whether they are reproducibly compatible. In addition, the CA genotype transmits grapevine red blotch virus (GRBV), but no information is available on the transmission capacity of the SE genotype. In this study, F1 offspring of mixed mating CA/SE *S. festinus* pairs exhibited varied physical characteristics compared to the offspring of the parental genotypes but acquired GRBV at similar rates. Likewise, the CA and SE parental genotypes acquired GRBV at similar rates, though the viral load in the salivary glands was significantly higher for SE than CA individuals and the transmission rate was significantly higher for the SE than the CA genotype. This is the first report of distinct GRBV transmission abilities of the two sexually compatible *S. festinus* populations, underscoring the need to study GRBV spread in the Southeastern United States.

**Abstract:**

Two phenotypically similar but genetically distinct genotypes of *Spissistilus festinus* (Say, 1830) (Hemiptera: Membracidae), a pest of legume crops in Southern United States and a vector of grapevine red blotch virus (GRBV) in California vineyards, exist. No information is available on whether the two *S. festinus* genotypes, i.e., California (CA) and Southeastern (SE), are sexually compatible or whether the SE genotype can transmit GRBV. In this study, we established mixed mating *S. festinus* pairs for which the F1 offspring varied phenotypically compared with the offspring of same genotype pairs but acquired GRBV isolate NY175 at similar rates (*p* = 0.96) and with a similar viral genome copy number (*p* = 0.34). Likewise, rates of GRBV acquisition were alike for the two parental CA (58%, 61/105) and SE (61%, 65/106) genotypes (*p* = 0.74), though the GRBV copy number in the salivary glands was overall significantly higher for SE than CA individuals (*p* = 0.02). Furthermore, the GRBV transmission rate was significantly higher for the SE genotype (89%, 16/18) than the CA genotype (50%, 8/16) (*p* = 0.04). These results revealed the existence of two sexually compatible *S. festinus* genotypes with distinct GRBV transmission abilities, suggesting the need to study GRBV ecology in Southeastern United States and areas where the two genotypes might co-exist.

## 1. Introduction

The three-cornered alfalfa hopper, *Spissistilus festinus* (Say, 1830) (Hemiptera: Membracidae), is an economically important pest of legume crops (peanut, alfalfa, soybean) in the Southern United States [1]. This treehopper is also of concern to the grape and wine industries in the United States for its ability to transmit grapevine red blotch virus (GRBV) in the vineyard [2]. GRBV is a member of the species *Grapevine red blotch virus* in the genus *Grablovirus,* of the family *Geminiviridae* [3,4]. GRBV is the causal agent of red blotch disease [5], which was first described in the mid-2000s in California [6,7]. Disease symptoms consist of foliar reddening on GRBV-infected, red-berried cultivars, and chlorosis and necrosis on GRBV-infected, white-berried cultivars [7,8,9]. Infected grapevines also experience delayed fruit ripening and reduced fruit quality [7,8,9,10,11,12]. Two distinct phylogenetic clades, i.e., 1 and 2, of GRBV isolates are found in cultivated and free-living grapevines [13,14,15,16].

*S. festinus* transmits GRBV in a circulative, non-propagative manner [17]. This means that the virus transits through the body of *S. festinus* but does not use the insect vector as a host for replication. The virus is first ingested by *S. festinus* feeding on the phloem sap of a GRBV-infected plant. Then, GRBV travels through the alimentary canal and crosses the gut of *S. festinus*. Next, the virus moves across the gut epithelium, circulates in the hemolymph, and reaches the salivary glands, which are connected to the salivary canal of *S. festinus*. Finally, GRBV is transmissible into the phloem of a healthy plant host upon feeding via the salivary canal. This transmission mode requires GRBV to move through the hemolymph-salivary gland barrier of *S. festinus* for acquisition and subsequent inoculation to occur [2,17]. 

A recent study indicated that there are two distinct genotypes of *S. festinus,* one residing in the Southeastern (SE) United States (Alabama, Mississippi, Georgia, North Carolina, and Virginia) and one in California (CA) [18]. Genetic analyses revealed up to 10.8% sequence divergence in the mitochondrial cytochrome C oxidase 1 (mt-COI) gene between specimens of the two phylogenetic clades of *S. festinus* that were grouped based on sampling location. These results were confirmed upon additional analysis of the internal transcribed spacer 2 region [18]. While genetically distinct, morphological differences are more subtle, with a slightly more elevated pronotum found in the SE specimens [18]. Genetic differences in a highly conserved mitochondrial gene and, to a lesser extent, morphological differences raise the question as to whether the two genotypes of *S. festinus* correspond to two divergent lineages of the same species or to two distinct species.

*S. festinus* is reportedly found across the United States, the Caribbean, and South America, though the northern and southern ranges of the species are not well described [1,19]. Much attention has been recently given to the CA genotype of *S. festinus* because of its role in the transmission of GRBV in CA vineyards [2], and amongst wine grape cultivars and free-living grapevines [6]. While the role of the CA genotype of *S. festinus* in secondary spread of GRBV is a recognized aspect in red blotch disease ecology, reciprocal research has not been conducted using the SE genotype. This knowledge gap merits attention, particularly since *S. festinus* has been found in states with red blotch diseased vineyards including Virginia [20], North Carolina [21], Tennessee [22], Florida [23], Texas [23], and Georgia [24,25]. In this work, we determined whether the two known genotypes of *S. festinus*, CA and SE, are lineages of the same species and able to mate, or two distinct species that are incapable of sexual reproduction. Furthermore, we characterized the GRBV transmission ability of the SE genotype of *S. festinus*. We hypothesized that the two *S. festinus* genotypes are two lineages of the same treehopper species, and that the SE genotype is capable of transmitting GRBV, likely with a different efficiency compared to the CA genotype. Here, we summarize our research on the establishment and characterization of *S. festinus* CA/SE mating pairs and their progeny, and on the transmission of GRBV by SE *S. festinus* specimens.

## 2. Materials and Methods

### 2.1. Plant Materials

Greenhouse-grown snapbean, *Phaseolus vulgaris* ‘Hystyle’, and alfalfa, *Medicago sativa* (Galaxy 100 Brand), were used as rearing plants for both *S. festinus* genotypes. Both *P. vulgaris* and *M. sativa* are leguminous plants that are preferred feeding hosts of *S. festinus* [26,27,28,29] and can be utilized in GRBV transmission assays in which *P. vulgaris* serves as GRBV donor and recipient material, and *M. sativa,* a nonhost of GRBV, is used in a *S. festinus* gut clearing step [17]. Both plant species were maintained in the greenhouse at 22 ± 3 °C and 16 h:8 h (light:dark photoperiod) with supplemental light as needed.

### 2.2. S. festinus Populations

*S. festinus* specimens of both genotypes were maintained in laboratory colonies on bean or alfalfa. For the CA genotype, morphologically and genetically identical insects [18] were collected from alfalfa fields in October of 2015 in Yolo County in CA, USA, November of 2015 from Fresno County, CA, USA, and annually from 2016 to 2019 from Yolo and San Joaquin Counties in CA, USA. These insects were combined to form a colony derived solely of the CA *S. festinus* genotype. 

For the SE genotype, insects were collected from bean plants in Pickens County in South Carolina, USA. These specimens were confirmed to be of the SE genotype using a diagnostic PCR assay [18] and then placed on bean plants within screened insect cages, as previously described [17]. Specimens from South Carolina were used in this study as representative of the SE genotype. 

The CA and SE colonies were maintained in separate controlled environmental chambers with rearing conditions set at 26 °C, 14:10 light/dark photoperiod, and 85% relative humidity, as previously described [2,17].

### 2.3. Establishment of Mixed Mating Pairs

Fourth and fifth instar nymphs of each *S. festinus* genotype were captured from the colonies and placed individually on detached *P. vulgaris* trifoliates using a small paint brush. Trifoliates consisting of the petiole and three leaflets and were placed in parafilm-covered 20 mL vials filled with distilled water through a premade hole. Vials and trifoliates were enclosed in a clear polypropylene 950 mL container (PK32T, Fabri-Kal, Kalamazoo, MI, USA) using a donut lid with a nylon screen (BugDorm 5002, MegaView Science, Taichung, Taiwan). Nymphs were observed daily, and sex was noted 24-h post-adult emergence. Sex determinations were made primarily by observing the presence or absence of an ovipositor on the underside of the insect, indicating female or male, respectively. 

Each mating pair was placed on new detached *P. vulgaris* trifoliates and constituted either individuals of matching or opposing *S. festinus* genotype pairs, i.e., ‘CA only’, ‘SE only’, ‘CA Male + SE Female’, or ‘SE Male + CA Female’. Mating pairs were created using adults that emerged withing 48 h of each other. This design ensured any adult encountered the opposite sex solely through the artificial selection of mating pairs. Due to this initial experimental design resulting in a disproportionately large number of emerging females for both genotypes (see Section 3), males of either genotype were selected from newly emerged adults in the growth chamber-maintained colonies. Mating pairs were observed for signs of oviposition, including swelling of the petiole and small incision-like cuts in the petiole, with and without visualizing eggs, and nymphal emergence.

Resultant nymphs were counted and moved to new detached *P. vulgaris* trifoliates based on their parental pair. The resulting adults were sexed and moved to full, caged *P. vulgaris* bean plants (BugDorm 6E610 Insect Rearing Cage, Taichung, Taiwan). After two weeks, select males and females were collected from each cage for phenotypic assessment and subsequent genetic characterization. The remaining insects were allowed to stay on the bean plants to mate and oviposit. These newly established colonies were maintained consistent with the CA and SE genotypes. 

### 2.4. Morphological Assessment of F1 Progeny of S. festinus Mixed Mating Pairs

Twelve *S. festinus* males and twelve *S. festinus* females were collected from the F1 generation of each mating pair and frozen for 20 min in a −80 °C freezer. Then, the length and height of each individual was measured using a SZX16 stereoscope (Olympus, Center Valley, PA, USA) and cellSense Standard software (version 1.18).

### 2.5. Genotype Determination of F1 Progeny of S. festinus Mixed Mating Pairs

A few F1 *S. festinus* individuals underwent diagnostic testing by PCR to ensure parental lineage. Diagnostic PCR primers designed to differentiate the *S. festinus* genotype targeted the mitochondrial cytochrome C oxidase 1 (mt-COI) gene [18]. Genomic DNA was isolated from *S. festinus* as described [18]. PCR products were resolved via agarose gel electrophoresis and post-staining by GelRed (Biotium, Fremont, CA, USA) followed by visualization under UV. When utilizing individuals of the SE genotype, a 145 bp PCR band is observed, while a 547 bp PCR band is obtained when testing individuals of the CA genotype [18].

### 2.6. GRBV Inoculation of P. vulgaris 

Two- to three-week old *P. vulgaris* plants were pinpricked via a sterile dissecting needle dipped in solid culture (Luria–Bertani medium supplemented with agar and kanamycin) of recombinant *Agrobacterium tumefaciens* C58C1 containing the GRBV isolate NY175 infectious clone corresponding to an isolate of phylogenetic clade 1 [5]. Pinpricks occurred along the petiole of each emerged trifoliate in 2 cm increments to ensure virus establishment [17]. Plants were allowed to incubate for one week and GRBV presence was confirmed in select petioles via diagnostic PCR. 

### 2.7. Acquisition of GRBV by S. festinus Populations

Virus acquisition by *S. festinus* was achieved on infected *P. vulgaris* plants after wrapping the mainstem in cheesecloth secured with a twist tie to ensure *S. festinus* only had access to GRBV-infected plant material. This step is crucial because *P. vulgaris* is a pseudo-systemic host of GRBV, and the virus is only present within the inoculated petioles and corresponding leaves [17]. A total of 100 *S. festinus* of each genotype were placed on wrapped *P. vulgaris* plants and allowed to feed for a six, eight, or ten-day acquisition access period (AAP), followed by a 48 h gut clearing period on alfalfa, a non-host of GRBV [17]. Then, select *S. festinus* specimens of each genotype were collected using a D-cell-powered aspirator (Gemplers, Janesville, WI, USA) and frozen at −20 °C for approximately 1 h. Next, heads with salivary glands were dissected under an SZX16 Olympus stereoscope (Center Valley, PA, USA) using sterile dissecting tweezers, as previously described [17]. *S. festinus* organs were stored individually at −20 °C for subsequent nucleic acid extractions and testing for GRBV by PCR. A gut clearing step was essential when assessing if specimens had acquired GRBV. Feeding on alfalfa cleared the stylet and mouthparts of GRBV such that if a dissected head and salivary gland tested positive for GRBV via PCR, it was due to the circulative movement of the virus to the salivary glands, not ingestion of GRBV-infected plant material. Acquisition experiments were replicated three times.

For the offspring of mixed *S. festinus* mating pairs, i.e., ‘CA Male + SE Female’ and ‘SE Male + CA Female’, 35 insects were allowed to feed for 12 days, a period shown to be sufficient for GRBV acquisition by the CA genotype [2,17,30], before being moved to alfalfa for 48 h. Insects were collected on alfalfa and dissections were conducted as previously described. 

### 2.8. Transmission Assays of GRBV by Both S. festinus Genotypes

Three hundred *S. festinus* individuals from the CA or SE genotype were allowed to feed on GRBV-infected *P. vulgaris* for a specific AAP and underwent gut clearing on alfalfa as described above. Then, *S. festinus* were moved to uninfected, detached *P. vulgaris* trifoliates in cohorts of five for a four-day inoculation access period (IAP), insects were collected from the detached chambers after the IAP. Next, inoculated petioles were collected ten days after *S. festinus* removal. Leaf and petiole tissues were processed for each trifoliate and stored in a −20 °C freezer for GRBV testing. Transmission assays were replicated twice.

### 2.9. Nucleic acid Extraction from Plant and S. festinus Tissues

Genomic DNA was isolated from *P. vulgaris* tissue and *S. festinus* using the MagMAX-96 AI/ND (ThermoFisher Scientific, Waltham, MA, USA) and the MagMAX Multi-Sample Ultra Kit (ThermoFisher Scientific, Waltham, MA, USA), respectively, on a KingFisher Flex or Apex instrument (ThermoFisher Scientific, Waltham, MA, USA). 

### 2.10. GRBV Detection and Quantification

GRBV presence in plant and insect tissues was determined via multiplex PCR using extracted genomic DNA and primer pairs designed to hybridize to the coat protein (CP) and replication-associated protein (RepA) open reading frames (ORF) of the GRBV genome [8,15]. PCR products were analyzed via agarose gel electrophoresis and UV illumination post-staining with GelRed (Biotium, Fremont, CA, USA). 

Select *S. festinus* head and salivary gland tissues containing GRBV, indicated via PCR, underwent further analysis via qPCR with primers designed in the RepA ORF and SYBR Green reagents [2,31]. The GRBV copy number was determined via the construction of a standard curve utilizing the recombinant plasmid pUC19 carrying the GRBV genome in *Escherichia coli* cells that were serially diluted to concentrations of 6 ng/µL, 60 pg/µL, 600 fg/µL, 6 fg/µL, 60 ag/µL, and 6 ag/µL [5] and the resultant C_T_ values.

### 2.11. Statistics

Statistical analyses were performed on the resultant nymphs of mixed mating pairs and sex of the emerged adults. Additional tests were conducted on the absolute DNA copy number quantification of GRBV in the heads and salivary glands of *S. festinus* individuals. Analyses of variance (ANOVA), proportion tests, and one-tailed *t*-tests were performed in the RStudio program (the R Project for Statistical Computing). The significance level was set at α = 0.05.

## 3. Results

### 3.1. Production of S. festinus Mating Pairs

Advanced nymphs of both *S. festinus* genotypes were placed on detached *P. vulgaris* trifoliates and allowed to emerge as adults. Emerging adults skewed female, accounting for most of the CA (82%, 23/28) and SE (86%, 24/28) adults. To account for the abundance of newly emerged females, newly emerged males from the original CA and SE colonies were used. This resulted in four parental mating pairs, including two mixed mating pairs: ‘CA Male + SE Female’ (*n* = 6), and ‘SE Male + CA Female’ (*n* = 5), and two mating pairs of the same genotype: ‘CA Male + CA Female’ (*n* = 5), referred to as ‘CA only’, and ‘SE Male + SE Female’ (*n* = 5) referred to as ‘SE only’.

### 3.2. The CA and SE S. festinus Genotypes Are Capable of Mating

**Figure 1:**
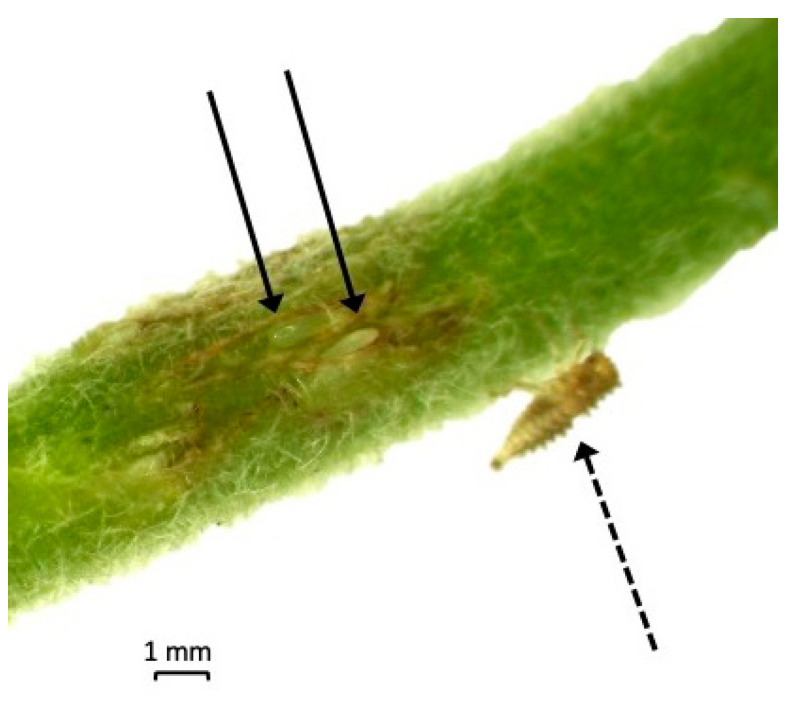
Oviposition by a female *Spissistilus festinus* individual of the California genotype with a first instar nymph on the petiole of a *Phaseolus vulgaris* trifoliate, indicated by the dashed arrow. Eggs are indicated by black arrows. The photo was taken under a SX16 Olympus stereoscope.

The four mating pairs were observed on *P. vulgaris* for signs of oviposition and nymphal emergence (Figure 1). The number of nymphs was recorded and moved to new detached trifoliates (Table 1). There was no difference in the fecundity and number of resulting nymphs, between the four parental pairs (*p* = 0.99) (Table 1).

**Figure 2:**
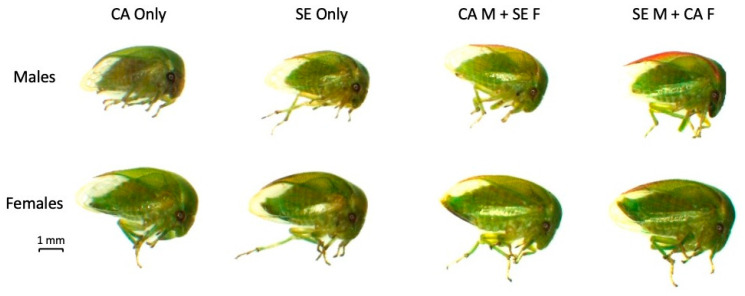
Representative male and female specimens of the F1 generation of mixed mating *Spissistilus festinus* pairs, including ‘CA only’, ‘SE only’, ‘CA Male + SE Female’, or ‘SE Male + CA Female’. Images were captured using a SX16 Olympus stereoscope.

The nymphs were allowed to emerge as adults and those of the two mixed mating pairs were sexed (Figure 2). The resultant adults of the ‘CA Male + SE Female’ and the ‘SE Male + CA Female’ mixed mating pairs skewed male (Table 2). Adults of each mixed mating pair were placed in separate cages with full *P. vulgaris* plants and allowed to mate and oviposit freely. These cages were used to establish colonies of the progeny of the initial mixed mating pairs. The continued emergence of nymphs and subsequent adults suggested that the offspring of these mixed mating pairs were not sterile.

Twelve males and twelve females from the F1 generation from each of the four mating pairs were collected for genotypic characterization by PCR. Using diagnostic primers targeting the mt-COI region of *S. festinus*, all ‘CA Male + SE Female’ specimens depicted the 145 bp DNA band (100%, *n* = 24) corresponding to the maternal SE *S. festinus*, and all the ‘SE Male + CA Female’ specimens showed the 547 bp PCR amplicon (100%, *n* = 24) indicative of the maternal CA *S. festinus*. As expected, all ‘CA only’ *S. festinus* (100%, *n* = 24) and all ‘SE only’ *S. festinus* (100%, *n* = 24) were correctly categorized, indicated by the presence of a 547 or 145 bp PCR amplicon, respectively. Taken together, these data indicated the two genotypes of *S. festinus* are capable of mating, resulting in fertile offspring. 

### 3.3. S. festinus F1 Generations Vary Phenotypically

**Figure 3:**
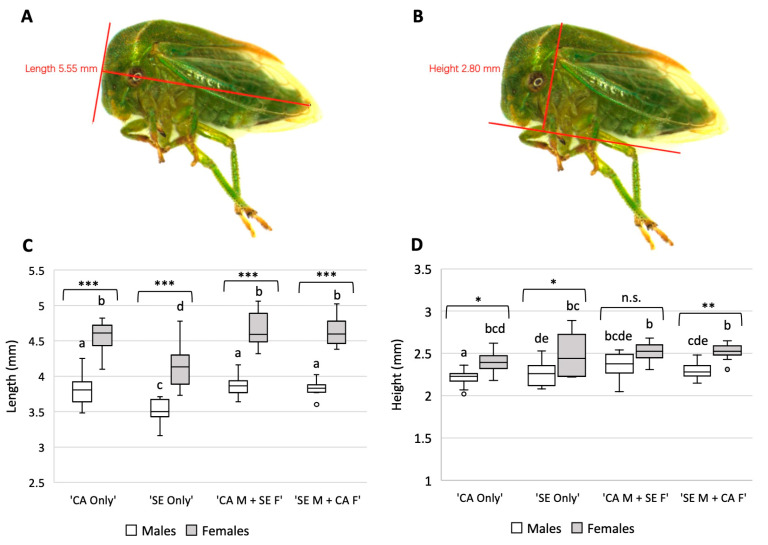
Measurements of F1 generation of *Spissistilus festinus* grouped by sex and parental pair. (**A**), Length measurement from the head to anus of a female CA *S. festinus*. (**B**), Height measurement from the mouthparts to the pronotum of a female CA *S. festinus*. All measurements were taken under a SX16 Olympus stereoscope indicated by the red lines artificially added using the perpendicular measuring tool on the cellSense Standard software (version 1.18). (**C**), Length in mm of *S. festinus* individuals. (**D**), Height in mm of *S. festinus* individuals. Brackets and asterisks represent statistical differences in measurements between males and females of the same parental pairs. Letters above the bars indicate statistically significant differences across all specimen groups as determined by ANOVA (*p* < 0.05). Each category consists of measurements of 12 individuals. Vertical axes are set to mm. ns: not significant; * correspond to *p*-values < 0.05, ** to *p*-values < 0.01, and *** to *p*-values < 0.001.

Twelve males and twelve females of the F1 generation from each of the four mating pairs were collected for phenotypic assessments (Figure 3A,B). The length of the specimens was significantly different (*p* < 0.001) between males and females of each parent type (Figure 3C) (Table S1), with longer males of the same parental type (*p* < 0.001) (Table S2). Similarly, all females differed from males regardless of the parental pair (Figure 3C). Additionally, adult height was significantly different amongst males and females of each parent pair (*p* < 0.001) (Figure 3D). Furthermore, females were taller than males of the same parental type (Table S3), with the exception of male and female progeny of the ‘CA Males + SE Females’ (*p* = 1.000). These data indicated differences in the length and height between the progeny of the four mating pairs.

### 3.4. Mixed Mating Pairs Are Capable of Acquiring GRBV

Preliminary work determined that the qPCR assay was capable of quantifying GRBV found within the salivary glands of *S. festinus*. Serial dilutions of pUC19 containing the genome of GRBV isolate NY358 produced a line of best fit that was used to determine the absolute amount of virus within the heads and salivary glands of *S. festinus* individuals (Figure S1).

**Figure 4:**
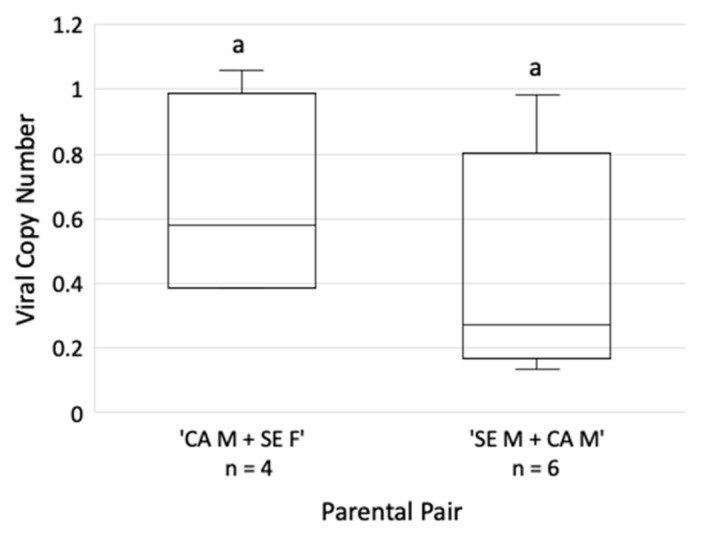
Copy number of the grapevine red blotch virus (GRBV) genome in the heads and salivary glands of dissected *Spissistilus festinus* individuals derived from mixed genotype parents after a 12-day acquisition access period and 48 h gut clearing on *Medicago sativa.* Letters above the bars indicate statistically significant differences as determined by *t*-test (*p* < 0.05). The number (*n*) of specimens tested for each category is shown. Vertical axes are set on the logarithmic scale of viral copy number.

Specimens of the mixed mating pair colonies were allowed to feed on GRBV-infected *P. vulgaris* to assess their virus acquisition capabilities. Both the ‘CA Male + SE Female’ and ‘SE Male + CA Female’ colonies acquired GRBV at the same rate (25%, 4/16 and 32%, 6/19, respectively), as determined by end-point PCR. The rate of heads with salivary glands testing positive for GRBV after undergoing dissections was similar between the two groups (*p* = 0.96) as determined by end-point PCR. Similarly, the GRBV copy number in the heads with salivary glands was similar between the mixed mating pairs (*p* = 0.34), as shown by qPCR (Figure 4). These data confirmed the acquisition capabilities of progeny derived from mixed *S. festinus* parental pairs. 

### 3.5. The Rate of GRBV Acquisition Is Similar between the CA and SE S. festinus Genotypes

After completing the virus acquisition step following a gut clearing on alfalfa, the heads with salivary glands of *S. festinus* of the CA and SE genotypes were tested for GRBV by end-point PCR. Results revealed a high rate of virus acquisition: 58% (61/105) and 61% (65/106) for the CA and SE *S. festinus* genotypes, respectively (Table 3). Differences in the rates of virus acquisition between the two genotypes were not significant (*p* = 0.74). Similarly, there was no significant difference in the rate of GRBV acquisition by the CA genotype between the 6-day AAP (67%, 23/34) and the 8-day AAP (45%, 15/33) (*p* = 0.11), or between the 6-day AAP and the 10-day AAP (61%, 23/38) (*p* = 0.70) (Table 3). Furthermore, no significant difference was observed between GRBV acquisition rates of the 8-day AAP and 10-day AAP (*p* = 0.30). This trend was consistent with the SE genotype. No significant difference in the rate of GRBV acquisition was observed when comparing the number of heads with salivary glands that tested positive for GRBV after a 6-day AAP (64%, 28/44), and an 8-day AAP (61%, 19/31), (*p* = 0.19), and again when comparing the 6-day AAP and 10-day AAP (58%, 18/31) (*p* = 0.57) or the 8-day AAP and a 10-day AAP (*p* = 0.58) (Table 3). 

When comparing the rates of acquisition between the two *S. festinus* genotypes, no significant difference was observed at 6-day AAP (*p* = 0.90), 8-day AAP (*p* = 0.31), or 10-day AAP (*p* = 1.00). These data indicated that GRBV is similarly acquired by the two *S. festinus* genotypes. 

### 3.6. GRBV Titer in the Salivary Glands Varies with the S. festinus Genotype

**Figure 5:**
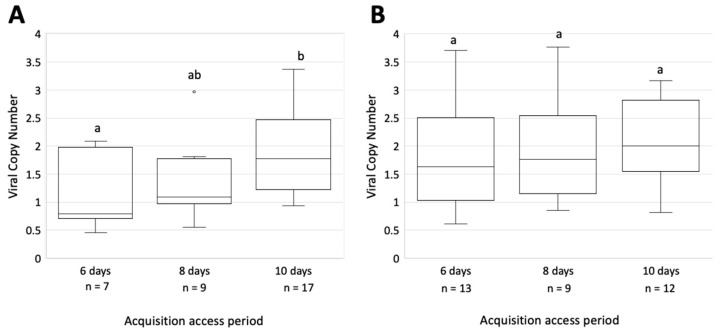
Copy number of the grapevine red blotch virus (GRBV) genome in the heads and salivary glands of dissected *Spissistilus festinus* individuals. (**A**), GRBV copy number in heads and salivary glands of the CA genotype following three acquisition access periods, i.e., 6, 8, and 10 days, and 48 h gut clearing on *Medicago sativa. (***B**), GRBV copy number in heads and salivary glands of *S. festinus* of the SE genotype. Letters above the bars indicate statistically significant differences as determined by ANOVA (*p* < 0.05). The number (*n*) of specimens tested for each category is shown. Vertical axes are set on the logarithmic scale of viral copy number.

GRBV was quantified by qPCR in heads and salivary glands of randomly selected *S. festinus* that tested positive for GRBV by end-point PCR to determine the amount of virus after acquisition. A difference in the number of viral copies in the heads and salivary glands of CA genotype specimens based on AAP was found, as shown by ANOVA (*p =* 0.03). Similarly, a significant difference in the viral copy number in the salivary glands of CA *S. festinus* specimens following a 6- and 10-day AAP (*p* = 0.047) was found by Tukey’s test (Figure 5). To the contrary, no difference in virus titer was found in the heads and salivary glands of SE genotype specimens when comparing any AAP (*p* = 0.81) (Figure 5). 

These results were further confirmed via one-tailed *t*-tests (Table S4). The GRBV copy number found in the CA genotype specimens were greatest following a 10-day AAP compared to the 8- and 6-day AAP (*p* = 0.04 and *p* = 0.01, respectively), but the 8-day AAP was not greater than the 6-day AAP (*p* = 0.25). These results were not observed amongst the heads and salivary glands of SE genotype specimens for which no difference was found across the three AAPs (Table S4). Interestingly, viral copy number in the head and salivary glands of SE specimens following a 10-day AAP was greater than that found in the head and salivary glands of CA specimens following an 8- or 6-day AAP (*p* = 0.02 and *p* = 0.01, respectively). Similarly, the GRBV copy number in SE specimens following a 6-day AAP was greater than that found in the CA specimens (*p* = 0.04). These data revealed that the GRBV copy number in the salivary glands is consistent in individuals of the SE genotype but increases during the acquisition period in individuals of the CA genotype. They also indicated that the GRBV titer is higher in the salivary glands of SE specimens compared to CA specimens.

### 3.7. S. festinus of the SE Genotype Transmit GRBV at a Higher Rate Than the CA Genotype

*S. festinus*-mediated transmission of GRBV from inoculated *P. vulgaris* plants to detached *P. vulgaris* trifoliates was observed for CA and SE genotypes at all AAPs (Table 4). Individuals from the CA genotype transmitted GRBV to 50% (8/16) of the detached bean trifoliates, while SE genotype specimens transmitted GRBV to 89% (16/18) of the detached bean trifoliates (Table 4). These rates were significantly different (*p* = 0.04). However, there was no significant difference between the rates of GRBV transmission between AAPs of the same genotype, or AAPs between genotypes (Table S5). These data revealed that the SE *S. festinus* genotype is more efficient at transmitting GRBV than the CA *S. festinus* genotype.

## 4. Discussion

In this study, we demonstrated that two genotypes of *S. festinus,* the CA and SE, are capable of mating and producing fertile offspring. These data indicated that the two populations are genotypes of the same species. Using fourth and fifth instar nymphs, artificial mating pairs were generated consisting of four different parental types, ‘CA only’, ‘SE only’, ‘CA Male + SE Female’, or ‘SE Male + CA Female’. Recording the number of emerged nymphs from each of the four pairs indicated no significant difference in fecundity (*p* = 0.99) (Table 1). 

Interestingly, the F1 generation of the mixed mating pairs skewed male (Table 2). These results are not altogether unexpected because this trend is observed in mixed biotype pairs of whiteflies (*Bemisia tabaci*) similarly resulting in a greater number of males compared to mating pairs of the same biotype [32,33]. Such mixed mating pairs also had no influence on the fecundity of mated females [33]. Of important note, using a diagnostic PCR to characterize a mt-COI region [18], the predicted 145 bp DNA target was amplified from all offspring of a SE female (100%, *n* = 48) and the predicted 547 bp DNA target was obtained from all offspring of a CA female (100%, *n* = 48). 

A wide range of resultant nymphs was recorded for the mating pairs (Table 1). This demonstrated the challenges associated with maintaining *S. festinus* in the detached chambers. The parafilm covering the water vial serves not only as a scaffold to hold the bean petiole straight, but also as a physical barrier to avoid nymphal drowning, though this was not an absolute solution for the tiny larvae. Furthermore, detached *P. vulgaris* trifoliates were often difficult to maintain in vials beyond two weeks. As such, nymphs were transferred to new trifoliates regularly, potentially causing undo stress. Nevertheless, individual *S. festinus* pairs were successful in mating and even capable of generating new colonies consisting of individuals originating from mixed mating pairs. 

Phenotypic assessment of the F1 generation demonstrated differences in the length and height of progeny between all four parent types (*p* < 0.001 for both) (Figure 3C,D). Importantly, we confirmed observations of an elevated pronotum observed in the SE genotype compared to the CA genotype (Table S2). This morphological difference was previously reported for the SE genotype [18]. Studies focusing on the virus acquisition capabilities of the mixed mating pair colonies determined that both acquire GRBV to the salivary glands at similar rates and GRBV quantities (Figure 4). Based on these results, we hypothesize that progeny of mixed parental pairs are capable of transmitting GRBV, as acquisition has proved essential to subsequent transmission of geminiviruses [34,35,36,37], including GRBV [17]. 

In this study, we documented, for the first time, the transmission of GRBV by the SE genotype. The rate of transmission by this genotype (89%, 16/18) is significantly higher than that of the CA genotype (50%, 8/16) (*p* = 0.04). Such differences in transmission efficiency have been observed in geminivirus transmission by in whiteflies, such as biotypes MED and MEAM1 in the acquisition, transmission, and retention of tomato chlorosis virus [38], and the transmission of tomato leaf curl Bangalore virus by the MEAM1 and Asia I biotypes [39]. We hypothesize that this difference in the rate of transmission is due to differences in the viral copy number of GRBV found in the heads of the two genotypes. The viral load in the heads with salivary glands of the SE genotype was consistent amongst the tested AAPs (*p* = 0.81) (Figure 5), and after a 10-day AAP was found to be greater than the GRBV copy number found in the salivary glands of CA individuals after an 8- and 6-day AAP (*p* = 0.02 and *p* = 0.01, respectively). The quantity of GRBV found in the salivary glands of CA specimens increased over time (Table S4). These data posit that increased GRBV presence in the salivary glands of *S. festinus* could potentially influence the rate of viral transmission. Additional research is required to test this hypothesis, determine the feeding behaviors of *S. festinus,* and assess GRBV interactions with *S. festinus* proteins or endosymbionts that could contribute to the greater GRBV acquisition efficiency of the SE genotype compared to the CA genotype. 

The inheritance of endosymbionts within the bacteriocytes is further called to question. Generally, endosymbionts are inherited vertically from the maternal parent via extracellular transit to the ovaries, or as in whiteflies, the transfer of entire bacteriocytes to the ovaries which become associated with the developing egg [40,41]. In the MEAM1 and MED biotypes of whiteflies, a single bacteriocyte is transferred to each oocyte and persists to subsequent generations [41,42]. This differs in a whitefly relative of *B. tabaci, Trialeurodes vaporariorum* in which several embryonic bacteriocytes are transferred to embryos and are likely being degraded within it [42]. To our knowledge such information is not known for treehopper species. No statistical difference was observed in the GRBV copy number found in the heads and salivary glands of mixed mating pair individuals after a 12-day AAP, despite the SE genotype acquiring GRBV more efficiently than the CA genotype (Table S4). Additional work is needed to determine endosymbionts that might aid in GRBV acquisition and the inheritance patterns of these bacterial species between the two *S. festinus* genotypes. 

The geographic range of *S. festinus* in the United States, and that of individual genotypes, is not well understood. The ability of the SE genotype and potential ability of *S. festinus* of mixed genotypic parentage to transmit GRBV is crucial to understanding red blotch disease ecology across the United States. Spread of GRBV has only been documented in California [8,43,44] and Southern Oregon [14,45], though the occurrence of the virus is reported in the Southeast [20,21,22,23,24,25] and Midwest [15,46,47,48]. Interestingly, GRBV has been found in free-living grapevines surrounding vineyards in California [13,16,49], Oregon [14], and Missouri [49], a fact likely due to insect mediated transmission.

In summary, we documented the potential for the two known genotypes of *S. festinus* to mate and produce fertile offspring. We noted phenotypic variations in the F1 generation of mixed mating pairs compared to offspring of same-genotype pairs. Colonies established from the F1 generation of individuals of mixed parental pairs are capable of GRBV acquisition, a fact not altogether surprising in light of both genotypes being capable of transmitting GRBV. Interestingly, the SE *S. festinus* genotype acquires GRBV more efficiently compared to the CA *S. festinus* genotype, likely contributing to increased rates of virus transmission. The potential for differential rates of GRBV transmission based on the *S. festinus* genotype highlights the need to study GRBV ecology in the Southeastern United States and regions where the two genotypes might co-exist.

## Figures and Tables

**Table 1:** Fecundity of individual *Spissistilus festinus* mating pairs on detached *Phaseolus vulgaris* trifoliates.

Parent Pair ^a^	Replicate ^b^	Observed ^c^	Average ^d^
CA Only	1	94	50.6
	2	35	
	3	58	
	4	43	
	5	23	
SE Only	1	1	54.2
	2	70	
	3	63	
	4	82	
	5	55	
CA Male + SE Female	1	55	52
	2	1	
	3	52	
	4	80	
	5	31	
	6	93	
SE Male + CA Female	1	3	46.8
	2	88	
	3	42	
	4	32	
	5	69	

^a^ Individual parental pair maintained on excised *P. vulgaris* trifoliates. ^b^ Detached chamber containing an individual *P. vulgaris* trifoliate. ^c^ Number of resultant nymphs. ^d^ Average number of resultant nymphs for each parental pair.

**Table 2:** Sex of adults in the F1 generation of mixed *Spissistilus festinus* mating pairs.

Parental Pair ^a^	Total ^b^	Average_T_ ^c^	Males	Average_M_^d^	Females	Average_F_^e^
CA Male + SE Female	106	17.7	59	9.8	47	7.8
SE Male + CA Female	51	10.2	30	12	21	4.2

^a^ Individual parental pair on individual *P. vulgaris* trifoliates. ^b^ Number of resultant adults across all detached trifoliates. ^c^ Average number of adults from each parent pair. ^d^ Average number of males from each parent pair. ^e^ Average number of females from each parent pair.

**Table 3:** Detection of grapevine red blotch virus (GRBV) in heads with salivary glands of two *Spissistilus festinus* genotypes after acquisition on infected *Phaseolus vulgaris* plants followed by a 48 h gut clearing step on *Medicago sativa*.

	Genotype ^a^		
Acquisition Access Period ^b^	CA ^c^	SE ^c^	Total ^d^
6	23/34 (68%)	28/44 (64%)	51/78 (65%)
8	15/33 (45%)	19/31 (61%)	34/64 (53%)
10	23/38 (61%)	18/31 (58%)	41/69 (59%)
Total ^e^	61/105 (58%)	65/106 (61%)	

^a^ Genotypes of *S. festinus.* ^b^ Acquisition access period (AAP) in days. ^c^ Proportion of heads and salivary glands testing positive for GRBV via multiplex PCR over total number tested. ^d^ Cumulative proportion of heads and salivary glands testing positive for GRBV based on AAP. ^e^ Cumulative proportion of heads and salivary glands testing positive for GRBV based on genotype.

**Table 4:** Transmission of grapevine red blotch virus (GRBV) by two *Spissistilus festinus* genotypes.

	Genotype ^a^		
Acquisition Access Period ^b^	CA ^c^	SE ^c^	Total ^d^
6	3/5 (60%)	5/5 (100%)	8/10 (80%)
8	3/5 (60%)	5/7 (71%)	8/12 (67%)
10	2/6 (33%)	6/6 (100%)	8/12 (67%)
Total ^e^	8/16 (50%)	16/18 (89%)	

^a^ Genotypes of *S. festinus*.^. b^ Acquisition access period (AAP) in days. ^c^ Proportion of *P. vulgaris* trifoliates testing positive for GRBV via PCR over total number tested. ^d^ Cumulative proportion of *P. vulgaris* trifoliates testing positive for GRBV based on AAP. ^e^ Cumulative proportion of *P. vulgaris* trifoliates testing positive for GRBV based on genotype.

## Data Availability

Raw data will be made available upon request.

## Supplementary Materials

The following supporting information can be downloaded at: https://www.mdpi.com/article/10.3390/insects14100831/s1, Figure S1: Resultant standard curves from serial dilutions of a GRBV NY358 monomer cloned into pUC19 from recombinant *Escherichia coli* DH5α cells used for absolute GRBV quantification from heads with salivary glands of *Spissistilus festinus* individuals; Table S1: Reported *p*-values determined by ANOVA detailing differences mm of the length and height of the F1 generation of *Spissistilus festinus* mating pairs; Table S2: Reported *p*-values determined by one-tailed *t*-test detailing differences in length of the F1 generation of the California (CA) and Southeast (SE) genotypes of *Spissistilus festinus* and the F1 generation mixed mating pairs; Table S3: Reported *p*-values determined by one-tailed *t*-test detailing differences in height of the F1 generation of the California (CA) and Southeast (SE) genotypes of *Spissistilus festinus* and the F1 generation mixed mating pairs; Table S4: Reported *p*-values determined by one-tailed *t*-test detailing differences in grapevine red blotch virus titer between heads and salivary glands of the California (CA) and Southeast (SE) genotypes of *Spissistilus festinus* after gut clearing on *Medicago sativa*; Table S5: Reported *p*-values determined by proportion tests detailing differences in the rate of grapevine red blotch virus transmission the California (CA) and Southeast (SE) genotypes of *Spissistilus festinus*.
