# Supplementary material for: Two Distinct Genotypes of Spissistilus festinus (Say, 1830) Reproduce and Differentially Transmit Grapevine Red Blotch Virus
Source: Insects. 2023 Oct 23;14(10):831. doi: 10.3390/insects14100831 (PMC10607809; doi:10.3390/insects14100831)
Supplement: Supplementary file 1 [file insects-14-00831-s001.zip › Supplemental_Genotype MS.docx]

**Table S1.** Reported *p-*values determined by ANOVA detailing differences mm of the length and height of the F1 generation of *Spissistilus festinus* mating pairs.

| Specimens 1^a^ | Specimens 2 | Length^b^ | Height^c^ |
| --- | --- | --- | --- |
| ‘CA Only’ Males | ‘CA Only’ Females | <0.001*** | 0.046* |
| ‘CA Only’ Males | ‘SE Only’ Males | 0.038* | 0.988 |
| ‘CA Only’ Males | ‘SE Only’ Females | 0.002** | <0.001*** |
| ‘CA Only’ Males | ‘CA M + SE F’ Males | <0.001*** | 0.188 |
| ‘CA Only’ Males | ‘CA M + SE F’ Females | <0.001*** | <0.001*** |
| ‘CA Only’ Males | ‘SE M + CA F’ Males | 1.000 | 0.800 |
| ‘CA Only’ Males | ‘SE M + CA F’ Females | <0.001*** | <0.001*** |
| ‘CA Only’ Females | ‘SE Only’ Males | <0.001*** | 0.326 |
| ‘CA Only’ Females | ‘SE Only’ Females | <0.001*** | 0.831 |
| ‘CA Only’ Females | ‘CA M + SE F’ Males | <0.001*** | 0.999 |
| ‘CA Only’ Females | ‘CA M + SE F’ Females | 0.919 | 0.360 |
| ‘CA Only’ Females | ‘SE M + CA F’ Males | <0.001*** | 0.735 |
| ‘CA Only’ Females | ‘SE M + CA F’ Females | 0.982 | 0.377 |
| ‘SE Only’ Males | ‘SE Only’ Females | <0.001 | 0.008 |
| ‘SE Only’ Males | ‘CA M + SE F’ Males | 0.004** | 0.698 |
| ‘SE Only’ Males | ‘CA M + SE F’ Females | <0.001*** | <0.001*** |
| ‘SE Only’ Males | ‘SE M + CA F’ Males | 0.018* | 0.998 |
| ‘SE Only’ Males | ‘SE M + CA F’ Females | <0.001*** | <0.001*** |
| ‘SE Only’ Females | ‘CA M + SE F’ Males | 0.024* | 0.468 |
| ‘SE Only’ Females | ‘CA M + SE F’ Females | <0.001*** | 0.995 |
| ‘SE Only’ Females | ‘SE M + CA F’ Males | 0.005** | 0.055 |
| ‘SE Only’ Females | ‘SE M + CA F’ Females | <0.001*** | 0.996 |
| ‘CA M + SE F’ Males | ‘CA M + SE F’ Females | <0.001*** | 0.113 |
| ‘CA M + SE F’ Males | ‘SE M + CA F’ Males | 0.999 | 0.966 |
| ‘CA M + SE F’ Males | ‘SE M + CA F’ Females | <0.001*** | 0.120 |
| ‘CA M + SE F’ Females | ‘SE M + CA F’ Males | <0.001*** | 0.005** |
| ‘CA M + SE F’ Females | ‘SE M + CA F’ Females | 0.999 | 1.000 |
| ‘SE M + CA F’ Males | ‘SE M + CA F’ Females | <0.001*** | 0.006** |

^a^Parental pair of *S. festinus.* ^b^*p*-values determined by measured lengths of *S. festinus* individuals. ^c^*p*-values determined by measured heights of *S. festinus* individuals. * denotes *p-*values < 0.05. ** denotes *p-*values < 0.01. *** denotes *p-*values < 0.001.

**Table S2.** Reported *p-*values determined by one-tailed t-test detailing differences in length of the F1 generation of the California (CA) and Southeast (SE) genotypes of *Spissistilus festinus* and the F1 generation mixed mating pairs*.*

| *x^a^* | $\bar{x}$^b^ | *y^a^* | $\bar{y}$ | *p-*value^c^ |
| --- | --- | --- | --- | --- |
| ‘CA Only’ Males | 3.796 | ‘CA Only’ Females | 4.562 | 1.000 |
| ‘CA Only’ Males | 3.796 | ‘SE Only’ Males | 3.522 | 0.001*** |
| ‘CA Only’ Males | 3.796 | ‘SE Only’ Females | 4.148 | 0.997 |
| ‘CA Only’ Males | 3.796 | ‘CA M + SE F’ Males | 3.860 | 0.800 |
| ‘CA Only’ Males | 3.796 | ‘CA M + SE F’ Females | 4.668 | 1.000 |
| ‘CA Only’ Males | 3.796 | ‘SE M + CA F’ Males | 3.818 | 0.621 |
| ‘CA Only’ Males | 3.796 | ‘SE M + CA F’ Females | 4.642 | 1.000 |
| ‘CA Only’ Females | 4.562 | ‘CA Only’ Males | 3.796 | <0.001*** |
| ‘SE Only’ Males | 3.522 | ‘CA Only’ Males | 3.796 | 0.999 |
| ‘SE Only’ Females | 4.148 | ‘CA Only’ Males | 3.796 | 0.003** |
| ‘CA M + SE F’ Males | 3.860 | ‘CA Only’ Males | 3.796 | 0.200 |
| ‘CA M + SE F’ Females | 4.668 | ‘CA Only’ Males | 3.796 | <0.001*** |
| ‘SE M + CA F’ Males | 3.818 | ‘CA Only’ Males | 3.796 | 0.379 |
| ‘SE M + CA F’ Females | 4.642 | ‘CA Only’ Males | 3.796 | <0.001*** |
| ‘CA Only’ Females | 4.562 | ‘SE Only’ Males | 3.522 | <0.001*** |
| ‘CA Only’ Females | 4.562 | ‘SE Only’ Females | 4.148 | 0.001*** |
| ‘CA Only’ Females | 4.562 | ‘CA M + SE F’ Males | 3.860 | <0.001*** |
| ‘CA Only’ Females | 4.562 | ‘CA M + SE F’ Females | 4.668 | 0.871 |
| ‘CA Only’ Females | 4.562 | ‘SE M + CA F’ Males | 3.818 | <0.001*** |
| ‘CA Only’ Females | 4.562 | ‘SE M + CA F’ Females | 4.642 | 0.830 |
| ‘SE Only’ Males | 3.522 | ‘CA Only’ Females | 4.562 | 1.000 |
| ‘SE Only’ Females | 4.148 | ‘CA Only’ Females | 4.562 | 0.999 |
| ‘CA M + SE F’ Males | 3.860 | ‘CA Only’ Females | 4.562 | 1.000 |
| ‘CA M + SE F’ Females | 4.668 | ‘CA Only’ Females | 4.562 | 0.129 |
| ‘SE M + CA F’ Males | 3.818 | ‘CA Only’ Females | 4.562 | 1.000 |
| ‘SE M + CA F’ Females | 4.642 | ‘CA Only’ Females | 4.562 | 0.170 |
| ‘SE Only’ Males | 3.522 | ‘SE Only’ Females | 4.148 | 1.000 |
| ‘SE Only’ Males | 3.522 | ‘CA M + SE F’ Males | 3.860 | 1.000 |
| ‘SE Only’ Males | 3.522 | ‘CA M + SE F’ Females | 4.668 | 1.000 |
| ‘SE Only’ Males | 3.522 | ‘SE M + CA F’ Males | 3.818 | 1.000 |
| ‘SE Only’ Males | 3.522 | ‘SE M + CA F’ Females | 4.642 | 1.000 |
| ‘SE Only’ Females | 4.148 | ‘SE Only’ Males | 3.522 | <0.001*** |
| ‘CA M + SE F’ Males | 3.860 | ‘SE Only’ Males | 3.522 | <0.001*** |
| ‘CA M + SE F’ Females | 4.668 | ‘SE Only’ Males | 3.522 | <0.001*** |
| ‘SE M + CA F’ Males | 3.818 | ‘SE Only’ Males | 3.522 | <0.001*** |
| ‘SE M + CA F’ Females | 4.642 | ‘SE Only’ Males | 3.522 | <0.001*** |
| ‘SE Only’ Females | 4.148 | ‘CA M + SE F’ Males | 3.860 | 0.006** |
| ‘SE Only’ Females | 4.148 | ‘CA M + SE F’ Females | 4.668 | 0.999 |
| ‘SE Only’ Females | 4.148 | ‘SE M + CA F’ Males | 3.818 | 0.003** |
| ‘SE Only’ Females | 4.148 | ‘SE M + CA F’ Females | 4.642 | 0.999 |
| ‘CA M + SE F’ Males | 3.860 | ‘SE Only’ Females | 4.148 | 0.994 |
| ‘CA M + SE F’ Females | 4.668 | ‘SE Only’ Females | 4.148 | <0.001*** |
| ‘SE M + CA F’ Males | 3.818 | ‘SE Only’ Females | 4.148 | 0.997 |
| ‘SE M + CA F’ Females | 4.642 | ‘SE Only’ Females | 4.148 | <0.001*** |
| ‘CA M + SE F’ Males | 3.860 | ‘CA M + SE F’ Females | 4.668 | 1.000 |
| ‘CA M + SE F’ Males | 3.860 | ‘SE M + CA F’ Males | 3.818 | 0.221 |
| ‘CA M + SE F’ Males | 3.860 | ‘SE M + CA F’ Females | 4.642 | 1.000 |
| ‘CA M + SE F’ Females | 4.668 | ‘CA M + SE F’ Males | 3.860 | <0.001*** |
| ‘SE M + CA F’ Males | 3.818 | ‘CA M + SE F’ Males | 3.860 | 0.779 |
| ‘SE M + CA F’ Females | 4.642 | ‘CA M + SE F’ Males | 3.860 | <0.001*** |
| ‘CA M + SE F’ Females | 4.668 | ‘SE M + CA F’ Males | 3.818 | <0.001*** |
| ‘CA M + SE F’ Females | 4.668 | ‘SE M + CA F’ Females | 4.642 | 0.390 |
| ‘SE M + CA F’ Males | 3.818 | ‘CA M + SE F’ Females | 4.668 | 1.000 |
| ‘SE M + CA F’ Females | 4.642 | ‘CA M + SE F’ Females | 4.668 | 0.610 |
| ‘SE M + CA F’ Males | 3.818 | ‘SE M + CA F’ Females | 4.642 | 1.000 |
| ‘SE M + CA F’ Females | 4.642 | ‘SE M + CA F’ Males | 3.818 | <0.001*** |

^a^*Spissistilus festinus* genotype parental pair and sex of cohort. ^b^The average length of each specimen measured in mm. ^c^p-value testing the alternative hypothesis: $\bar{x}-\bar{y}$ > 0. * denotes *p-*values < 0.05. ** denotes *p-*values < 0.01. *** denotes *p-*values < 0.001.

**Table S3.** Reported *p-*values determined by one-tailed t-test detailing differences in height of the F1 generation of the California (CA) and Southeast (SE) genotypes of *Spissistilus festinus* and the F1 generation mixed mating pairs*.*

| *x^a^* | $\bar{x}$^b^ | *y^a^* | $\bar{y}$ | *p-*value^c^ |
| --- | --- | --- | --- | --- |
| ‘CA Only’ Males | 2.212 | ‘CA Only’ Females | 2.392 | 0.999 |
| ‘CA Only’ Males | 2.212 | ‘SE Only’ Males | 2.262 | 0.832 |
| ‘CA Only’ Males | 2.212 | ‘SE Only’ Females | 2.475 | 0.998 |
| ‘CA Only’ Males | 2.212 | ‘CA M + SE F’ Males | 2.358 | 0.996 |
| ‘CA Only’ Males | 2.212 | ‘CA M + SE F’ Females | 2.518 | 1.000 |
| ‘CA Only’ Males | 2.212 | ‘SE M + CA F’ Males | 2.298 | 0.979 |
| ‘CA Only’ Males | 2.212 | ‘SE M + CA F’ Females | 2.517 | 1.000 |
| ‘CA Only’ Females | 2.392 | ‘CA Only’ Males | 2.212 | <0.001*** |
| ‘SE Only’ Males | 2.262 | ‘CA Only’ Males | 2.212 | 0.999 |
| ‘SE Only’ Females | 2.475 | ‘CA Only’ Males | 2.212 | 0.003** |
| ‘CA M + SE F’ Males | 2.358 | ‘CA Only’ Males | 2.212 | 0.200 |
| ‘CA M + SE F’ Females | 2.518 | ‘CA Only’ Males | 2.212 | <0.001*** |
| ‘SE M + CA F’ Males | 2.298 | ‘CA Only’ Males | 2.212 | 0.379 |
| ‘SE M + CA F’ Females | 2.517 | ‘CA Only’ Males | 2.212 | <0.001*** |
| ‘CA Only’ Females | 2.392 | ‘SE Only’ Males | 2.262 | 0.018* |
| ‘CA Only’ Females | 2.392 | ‘SE Only’ Females | 2.475 | 0.843 |
| ‘CA Only’ Females | 2.392 | ‘CA M + SE F’ Males | 2.358 | 0.284 |
| ‘CA Only’ Females | 2.392 | ‘CA M + SE F’ Females | 2.518 | 0.992 |
| ‘CA Only’ Females | 2.392 | ‘SE M + CA F’ Males | 2.298 | 0.036* |
| ‘CA Only’ Females | 2.392 | ‘SE M + CA F’ Females | 2.517 | 0.994 |
| ‘SE Only’ Males | 2.262 | ‘CA Only’ Females | 2.392 | 0.982 |
| ‘SE Only’ Females | 2.475 | ‘CA Only’ Females | 2.392 | 0.157 |
| ‘CA M + SE F’ Males | 2.358 | ‘CA Only’ Females | 2.392 | 0.716 |
| ‘CA M + SE F’ Females | 2.518 | ‘CA Only’ Females | 2.392 | 0.009** |
| ‘SE M + CA F’ Males | 2.298 | ‘CA Only’ Females | 2.392 | 0.964 |
| ‘SE M + CA F’ Females | 2.517 | ‘CA Only’ Females | 2.392 | 0.008** |
| ‘SE Only’ Males | 2.262 | ‘SE Only’ Females | 2.475 | 0.991 |
| ‘SE Only’ Males | 2.262 | ‘CA M + SE F’ Males | 2.358 | 0.939 |
| ‘SE Only’ Males | 2.262 | ‘CA M + SE F’ Females | 2.518 | 1.000 |
| ‘SE Only’ Males | 2.262 | ‘SE M + CA F’ Males | 2.298 | 0.755 |
| ‘SE Only’ Males | 2.262 | ‘SE M + CA F’ Females | 2.517 | 1.000 |
| ‘SE Only’ Females | 2.475 | ‘SE Only’ Males | 2.262 | 0.009** |
| ‘CA M + SE F’ Males | 2.358 | ‘SE Only’ Males | 2.262 | 0.061 |
| ‘CA M + SE F’ Females | 2.518 | ‘SE Only’ Males | 2.262 | <0.001*** |
| ‘SE M + CA F’ Males | 2.298 | ‘SE Only’ Males | 2.262 | 0.245 |
| ‘SE M + CA F’ Females | 2.517 | ‘SE Only’ Males | 2.262 | <0.001*** |
| ‘SE Only’ Females | 2.475 | ‘CA M + SE F’ Males | 2.358 | 0.086 |
| ‘SE Only’ Females | 2.475 | ‘CA M + SE F’ Females | 2.518 | 0.711 |
| ‘SE Only’ Females | 2.475 | ‘SE M + CA F’ Males | 2.298 | 0.018* |
| ‘SE Only’ Females | 2.475 | ‘SE M + CA F’ Females | 2.517 | 0.707 |
| ‘CA M + SE F’ Males | 2.358 | ‘SE Only’ Females | 2.475 | 0.914 |
| ‘CA M + SE F’ Females | 2.518 | ‘SE Only’ Females | 2.475 | 0.289 |
| ‘SE M + CA F’ Males | 2.298 | ‘SE Only’ Females | 2.475 | 0.982 |
| ‘SE M + CA F’ Females | 2.517 | ‘SE Only’ Females | 2.475 | 0.293 |
| ‘CA M + SE F’ Males | 2.358 | ‘CA M + SE F’ Females | 2.518 | 1.000 |
| ‘CA M + SE F’ Males | 2.358 | ‘SE M + CA F’ Males | 2.298 | 0.221 |
| ‘CA M + SE F’ Males | 2.358 | ‘SE M + CA F’ Females | 2.517 | 1.000 |
| ‘CA M + SE F’ Females | 2.518 | ‘CA M + SE F’ Males | 2.358 | <0.001*** |
| ‘SE M + CA F’ Males | 2.298 | ‘CA M + SE F’ Males | 2.358 | 0.779 |
| ‘SE M + CA F’ Females | 2.517 | ‘CA M + SE F’ Males | 2.358 | <0.001*** |
| ‘CA M + SE F’ Females | 2.518 | ‘SE M + CA F’ Males | 2.298 | <0.001*** |
| ‘CA M + SE F’ Females | 2.518 | ‘SE M + CA F’ Females | 2.517 | 0.483 |
| ‘SE M + CA F’ Males | 2.298 | ‘CA M + SE F’ Females | 2.518 | 1.000 |
| ‘SE M + CA F’ Females | 2.517 | ‘CA M + SE F’ Females | 2.518 | 0.517 |
| ‘SE M + CA F’ Males | 2.298 | ‘SE M + CA F’ Females | 2.517 | 1.000 |
| ‘SE M + CA F’ Females | 2.517 | ‘SE M + CA F’ Males | 2.298 | <0.001*** |

^a^*Spissistilus festinus* genotype parental pair and sex of cohort. ^b^The average height of each specimen measured in mm. ^c^p-value testing the alternative hypothesis: $\bar{x}-\bar{y}$ > 0. * denotes *p-*values < 0.05. ** denotes *p-*values < 0.01. *** denotes *p-*values < 0.001.

**Table S4.** Reported *p-*values determined by one-tailed t-test detailing differences in grapevine red blotch virus titer between heads and salivary glands of the California (CA) and Southeast (SE) genotypes of *Spissistilus festinus* after gut clearing on *Medicago sativa.*

| *x^a^* | $\bar{x}$^b^ | *y^a^* | $\bar{y}$ | *p-*value^c^ |
| --- | --- | --- | --- | --- |
| 10-CA | 1.964 | 10-SE | 2.070 | 0.642 |
| 10-CA | 1.964 | 8-CA | 1.386 | 0.034* |
| 10-CA | 1.964 | 8-SE | 1.949 | 0.483 |
| 10-CA | 1.964 | 6-CA | 1.146 | 0.010** |
| 10-CA | 1.964 | 6-SE | 1.840 | 0.353 |
| 10-SE | 2.070 | 10-CA | 1.964 | 0.358 |
| 8-CA | 1.386 | 10-CA | 1.964 | 0.946 |
| 8-SE | 1.949 | 10-CA | 1.964 | 0.517 |
| 6-CA | 1.146 | 10-CA | 1.964 | 0.990 |
| 6-SE | 1.840 | 10-CA | 1.964 | 0.646 |
| 10-SE | 2.070 | 8-CA | 1.386 | 0.024* |
| 10-SE | 2.070 | 8-SE | 1.949 | 0.375 |
| 10-SE | 2.070 | 6-CA | 1.146 | 0.007** |
| 10-SE | 2.070 | 6-SE | 1.840 | 0.256 |
| 8-CA | 1.386 | 10-SE | 2.070 | 0.976 |
| 8-SE | 1.949 | 10-SE | 2.070 | 0.624 |
| 6-CA | 1.146 | 10-SE | 2.070 | 0.993 |
| 6-SE | 1.840 | 10-SE | 2.070 | 0.743 |
| 8-CA | 1.386 | 8-SE | 1.949 | 0.917 |
| 8-CA | 1.386 | 6-CA | 1.146 | 0.247 |
| 8-CA | 1.386 | 6-SE | 1.840 | 0.890 |
| 8-SE | 1.949 | 8-CA | 1.386 | 0.083 |
| 6-CA | 1.146 | 8-CA | 1.386 | 0.754 |
| 6-SE | 1.840 | 8-CA | 1.386 | 0.110 |
| 8-SE | 1.949 | 6-CA | 1.146 | 0.030 |
| 8-SE | 1.949 | 6-SE | 1.840 | 0.396 |
| 6-CA | 1.146 | 8-SE | 1.949 | 0.970 |
| 6-SE | 1.840 | 8-SE | 1.949 | 0.604 |
| 6-CA | 1.146 | 6-SE | 1.840 | 0.963 |
| 6-SE | 1.840 | 6-CA | 1.146 | 0.037* |

^a^Acquisition access period and *Spissistilus festinus* genotype head with salivary glands. ^b^The mean log transformation of viral copy number determined via qPCR. ^c^p-value testing the alternative hypothesis: $\bar{x}-\bar{y}$ > 0. * denotes *p-*values < 0.05. ** denotes *p-*values < 0.01. *** denotes *p-*values < 0.001.

**Table S5.** Reported *p-*values determined by proportion tests detailing differences in the rate of grapevine red blotch virus transmission the California (CA) and Southeast (SE) genotypes of *Spissistilus festinus.*

| x^a^ | GRBV_x_^b^ | y^a^ | GRBV_y_ | p-value^c^ |
| --- | --- | --- | --- | --- |
| 10-CA | 2/6 | 8-CA | 3/5 | 0.782 |
| 10-CA | 2/6 | 6-CA | 3/5 | 0.782 |
| 10-CA | 2/6 | 10-SE | 6/6 | 0.066 |
| 10-CA | 2/6 | 8-SE | 5/7 | 0.415 |
| 10-CA | 2/6 | 6-SE | 5/5 | 0.097 |
| 8-CA | 3/5 | 6-CA | 2/6 | 0.782 |
| 8-CA | 3/5 | 10-SE | 6/6 | 0.354 |
| 8-CA | 3/5 | 8-SE | 5/7 | 1.000 |
| 8-CA | 3/5 | 6-SE | 5/5 | 0.429 |
| 6-CA | 3/5 | 10-SE | 6/6 | 0.354 |
| 6-CA | 3/5 | 8-SE | 5/7 | 1.000 |
| 6-CA | 3/5 | 6-SE | 5/5 | 0.429 |
| 10-SE | 6/6 | 8-SE | 5/7 | 0.514 |
| 10-SE | 6/6 | 6-SE | 5/5 | NA |
| 8-SE | 5/7 | 6-SE | 5/5 | 0.601 |
| Total-CA | 8/16 | Total-SE | 16/18 | 0.035* |

^a^Acquisition access period and *Spissistilus festinus* genotype head with salivary glands. ^b^Proportion of *P. vulgaris* trifoliates testing positive for GRBV via PCR over total number tested. ^c^p-value testing the alternative hypothesis: GRBV_x_ $\neq$ GRBV_y._ * denotes *p-*values < 0.05. ** denotes *p-*values < 0.01. *** denotes *p-*values < 0.001.


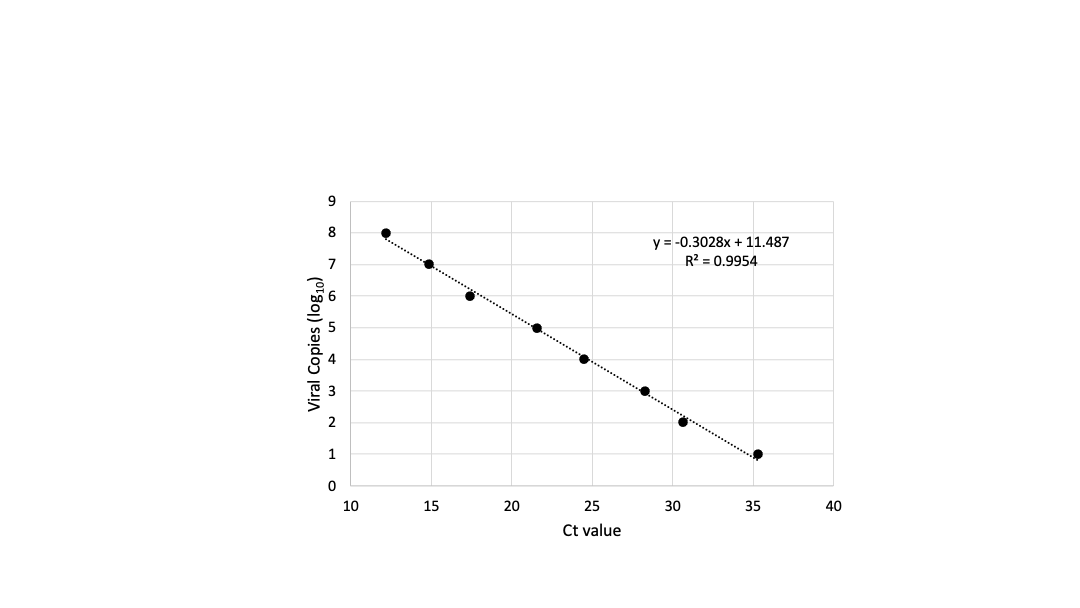
**Figure S1**. Resultant standard curves from serial dilutions of a GRBV NY358 monomer cloned into pUC19 from recombinant *Escherichia coli* DH5α cells used for absolute GRBV quantification from heads with salivary glands of *Spissistilus festinus* individuals.
