# Supplementary figures and images for: Two Distinct Genotypes of Spissistilus festinus (Say, 1830) Reproduce and Differentially Transmit Grapevine Red Blotch Virus
Source: Insects. 2023 Oct 23;14(10):831. doi: 10.3390/insects14100831 (PMC10607809; doi:10.3390/insects14100831)

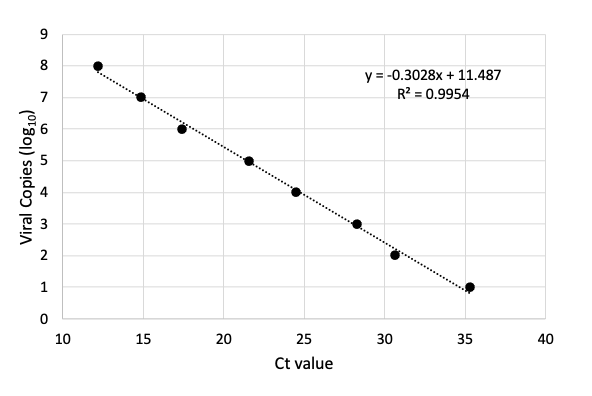

Supplement: Supplementary file 1 [file insects-14-00831-s001.zip › SupFig1.tiff]
